# Risk, Outcomes, and Trends of Clostridium Difficile Infection in Multiple Myeloma Patients from a Nationwide Analysis

**DOI:** 10.7759/cureus.4391

**Published:** 2019-04-05

**Authors:** Dan Ran-Castillo, Adegbala Oluwole, Munder Abuaisha, Angela R Banks Paulino, Ahmad Alkhatatneh, Jeehoon Jang, Sahai Donaldson, Jonathan Shammash, Karlene Williams

**Affiliations:** 1 Internal Medicine, Englewood Hospital, Englewood, USA

**Keywords:** multiple myeloma, risk and outcome, trend, clostridium difficile infection (cdi)

## Abstract

Background: Patients hospitalized with hematologic malignancy are particularly vulnerable to infection. We sought to determine the risk of Clostridium difficile infection (CDI) in hospitalization with multiple myeloma (MM), as well as its outcomes and trends, using a nationally representative database.

Methods: The Nationwide Inpatient Sample (NIS) from January 2010 to September 2015 was used for this study. We identified all patients aged 18 years or older with a diagnosis of MM using the International Classification of Disease, Ninth Revision, Clinical Modification (ICD-9-CM) codes. We identified trends in the annual rates of CDI in MM using negative binomial regressions with robust error variance. We conducted multivariate logistic regression to determine the incidence and the associated risk factors of CDI in MM and compared the outcomes between those with and without CDI using the propensity score method inverse probability weighting to adjust for baseline covariates.

Results: In our cohort study of 114,249 MM patients, 45.96% were females and 54.04% were males. CDI was present in 3.1% of the MM patients. The number of CDI cases increased over the study period with an average rate of 3.27% per year. The mortality rate decreased over the same period with an average rate of 10% decrease per year. Hematopoietic stem cell transplantation (HSCT), neutropenia, inflammatory disease, atrial fibrillation (AF), and chronic kidney disease (CKD) were significant associated risk factors of CDI in MM patients. After adjusting for covariates, patients with CDI had a prolonged hospital stay, inpatient mortality, and significantly increased odds of acute kidney injury (AKI) and AKI requiring hemodialysis, along with higher healthcare resources utilization with significantly higher hospital costs.

Conclusion: MM patients with CDI have significantly increased odds of inpatient mortality, AKI, and AKI requiring hemodialysis. They also have increased healthcare resource utilization compared with those without CDI. Despite the increased rate of the CDI over the years, the mortality rate is going down.

## Introduction

Clostridium difficile infections (CDI) have become the most common cause of healthcare-associated infections in the United States (US) hospitals, and the excess healthcare costs related to CDI are estimated to be as much as 4.8 billion US dollars for acute care facilities alone [1]. Patients admitted for hematopoietic malignancy have an increased risk of CDI, and multiple risk factors have been reported, such as chemotherapy and hematopoietic stem cell transplantation (HSCT) [2]. Multiple myeloma (MM) accounts for more than 17% of hematologic malignancies in the US [3]. To our knowledge, no large sample study evaluating the association between MM and CDI has been reported. Furthermore, in a recent cohort study, CDI was reported as the most common bacterial infection, occurring in one-third of those patients post-HSCT [2]. CDI most often occurred before engraftment, suggesting that neutropenia, more intense exposure to antimicrobials, immunosuppression, and transmission in the hospital environment are likely to be major risk factors. The risk factors and the outcomes of CDI in patients with hematopoietic malignancies, such as leukemia and lymphoma, have been well-documented [4]; a single center study has shown that levofloxacin prophylaxis in MM patients undergoing autologous HSCT increased the risk of CDI [5]. However, only limited data have assessed the incidence, risk factors, and outcomes of CDI in patients with MM. This study evaluates the incidence, associated risk factors, outcomes, and trends of CDI in a cohort of MM patients utilizing the largest all-payer, inpatient database in the US.

## Materials and methods

This was a cross-sectional study of hospital admissions for patients with a diagnosis of MM between 2010 and September 2015. This study was conducted using the Nationwide Inpatient Sample (NIS) of the Healthcare Cost and Utilization Project (HCUP) sponsored by the Agency for Healthcare Research and Quality (AHRQ). The NIS is a yearly survey of 20% of every admission from more than 4,000 hospitals across 30 states in the US and the District of Columbia [6]. The NIS has been validated in numerous studies to provide reliable estimates of admissions within the US [7-8]. Methods of data collection and administration of the NIS are detailed by the HCUP [9]. This study was considered exempt from Institutional Review Board approval of EHMC because the Healthcare Cost and Utilization Project-NIS dataset contain de-identified patient information.

We identified hospitalizations for adults aged 18 years or older with a discharge diagnosis of multiple myeloma using the International Classification of Diseases, Ninth Revision, Clinical Modification (ICD-9-CM) codes. We performed a comparative analysis of the outcomes of multiple myeloma patients with or without CDI on admission. Our primary outcome was in-hospital mortality. Secondary outcomes included the length of stay, hospitalization costs, acute kidney injury, and acute kidney injury requiring dialysis. A list of ICD-9-CM codes used to define clinical outcomes and comorbidities is included in Table 1. We estimated the cost of each inpatient stay by multiplying the total hospital charges with cost-to-charge ratios [10]. To account for the effect of inﬂation on hospital charges, we used data from the Bureau of Labor Statistic’s medical care component of the Consumer Price Index (CPI) and presented the data in 2017 US dollars [11].

**Table 1:** International Classification of Disease, Ninth Revision, Clinical Modification (ICD-9-CM) Codes CCS: Clinical Classifications Software

Variables	ICD-9-CM diagnostic/procedural codes and CCS codes
Patient Population	
Multiple myeloma	203.0; 203; 203.00; 203.02
Clostridium difficile infection	008.45
Comorbidities	
Prior cerebrovascular disease	V12.54; 438.x
Coronary artery disease	412; 414.00 - 414.07; 414.2; 414.3; 414.8; 414.9
Congestive heart failure	402.01; 402.11; 402.91; 404.01; 404.03; 404.11; 404.13; 404.91; 404.93; 428
Smoking	V1582 and 305.1
Dyslipidemia	CCS code 53
Prior myocardial infarction	V45.82
Prior percutaneous coronary intervention	412
Prior coronary artery bypass grafting	V45.81
Prior pacemaker	V45.01
Stem cell transplantation	V42.82; V42.81
Neutropenia	288.0; 28801; 28802; 288.03; 28804; 284.1
Chemotherapy	V58.1; V58.11; V67.2; V87.41
Inflammatory bowel disease	555.9; 560.89; 556.8; 556
In-hospital Clinical Outcome	
Renal complications	
Acute kidney injury	584.5 to 584.9
Hemodialysis	39.85
Acute kidney injury requiring dialysis	584.5 to 584.9, plus 39.85

We used the following as baseline characteristics: demographics (age, sex, race, primary expected payer, and median household income for the patient’s ZIP code), hospital-level characteristics (region, bed size, location, teaching status), and relevant comorbidities. The severity of comorbidities was quantified using the Elixhauser comorbidity index (ECI) [12].

Statistical analysis

All the data extraction and analyses were done with the Statistical Analysis System (SAS), V.9.4 (SAS Institute Inc., Cary, NC), and P-values are two-sided with a significance threshold of < 0.05. The Bonferroni corrected p-value was reported for multiple comparisons. We used absolute standardized differences (ASD) to compare the baseline characteristics. ASD (calculated as the differences in means or proportions divided by a pooled estimated of the standard deviation (SD)) is not as sensitive to sample size compared with traditional significance testing and hence, is useful in identifying clinically meaningful differences. An ASD of > 0.1 is considered clinically meaningful [13].

To minimize the effect of confounding and obtain an unbiased estimate of the effect of CDI on clinical outcomes, differences in baseline characteristics of the patients with or without CDI were balanced using propensity score methods to estimate the average effect of CDI on clinical outcomes. First, we conducted multivariate logistic regression to estimate the propensity score representing the probability of developing CDI based on an individual’s covariates. The following data were not available for a percentage of patients: race (6.31%), primary expected payer (0.19%), socioeconomic status (2.02%), hospital bed size (0.29%), and teaching hospital status (0.29%). We excluded the patients with the missing observations from the model given that observations were missing at random, except for race, where the missing observations were replaced with the dominant category. The propensity scores were used to calculate the inverse probability of CDI-weighted estimations for each patient included in the study [14]. For inverse probability weighing regression, individuals with CDI were weighted by the reciprocal of their propensity score, and individuals without CDI were weighted by one, minus the reciprocal of their propensity score. Once the weights were applied, the balance was assessed by examining standardized differences between the two groups [15].

We used generalized estimating equations with an exchangeable working correlation matrix accounting for clustering of outcomes within the hospital to determine the factors associated with developing CDI in MM. The model included patient-level variables, such as age, sex, and comorbidities, as well as hospital-level variables, such as hospital size (number of beds), hospital region, and teaching status. Choice of covariates for the multivariate analyses was based on the plausibility that they could be associated with CDI. For outcome computation after the propensity score method, binary outcomes, including inpatient mortality, were computed with binomial logistic regressions. For the discrete numeric overdispersed count distribution variable (the length of stay), the generalized linear model regressions with log link function with negative binomial distribution were used; total hospital cost, a continuous variable, generalized linear model regressions with a gamma distribution, and log link function were employed. We reported the odds ratio (OR) for our binary outcome and mean ratios (MR) for the numeric outcomes. We evaluated a trend in mortality by fitting a Poisson regression model with a robust error variance to evaluate for changes in CDI per year (adjusting for demographics, comorbidities, and hospital characteristics) and inserting the year variable into the model assuming the association to be linear.

As recommended by HCUP, analyses were performed in SAS with appropriate statements to account for the complex clustered sampling methodology [10, 16].

## Results

Table 2 shows the baseline characteristics of MM hospitalizations by CDI. An estimated 561,384 hospitalizations with MM were identified. Participants were predominantly white (65.25%) and males. They had mostly been admitted to urban teaching hospitals, and a large percentage of patients had an Elixhauser comorbidity score of 4 or more. Of these, 3.11 % had CDI. Compared with patients without CDI, patients with CDI were more likely to have CKD, AF, HSCT, neutropenia, inflammatory bowel disease, and a higher comorbidity index.

**Table 2:** Baseline Characteristics of Participants with Multiple Myeloma by Clostridium difficile (C. diff) in the United States #ASD: number of absolute standardized differences

	Multiple Myeloma		Total	#ASD
	Yes	No		
No. of observation unweighted	3,549 (3.11%)	110,700 (96.89%)	114,249	
No. of observation weighted	17,470	543,915	561,384	
Age categories				0.09
18-49	5.42	5.80	5.79	
50-59	14.97	15.67	15.64	
60-69	31.38	27.88	27.99	
70-79	29.97	29.16	29.18	
≥ 80	18.26	21.50	21.39	
Female	48.67	45.87	45.96	0.06
Race/Ethnicity				0.05
White	67.02	65.20	65.25	
Black	21.13	22.41	22.37	
Hispanic	7.40	7.66	7.65	
#Others	4.45	4.74	4.73	
Comorbidities				
Dyslipidemia	25.77	29.22	29.11	0.07
Atrial fibrillation	21.60	17.32	17.47	0.11
Chronic obstructive lung disease	17.21	16.69	16.71	0.01
Hypertension	59.22	58.82	58.83	0.01
Peripheral vascular diseases	5.45	4.84	4.85	0.03
Stem cell transplantation	13.94	9.88	10.01	0.13
Neutropenia	12.75	7.63	7.79	0.17
Chemotherapy	7.81	8.23	8.22	0.02
Inflammatory bowel disease	4.38	2.59	2.64	0.10
Diabetes	25.37	25.39	25.39	0.00
Obesity	7.30	7.37	7.39	0.00
Deficiency anemia	40.30	38.83	38.88	0.03
Congestive heart failure	19.56	20.99	19.60	0.04
Chronic renal disease	38.30	31.09	31.31	0.15
Liver disease	2.20	2.30	2.29	0.01
Elixhauser score				0.23
0	4.77	7.62	7.53	
1 - 3	25.70	33.93	33.67	
≥ 4	69.52	58.56	58.80	
Hospital bed size				0.07
Small	11.03	12.84	12.78	
Medium	21.87	23.36	23.31	
Large	67.09	63.80	63.90	
Hospital teaching status				0.10
Rural	5.58	8.26	8.17	
Urban, non-teaching	27.88	30.31	30.23	
Urban, teaching	66.54	61.44	61.60	
Expected primary payer				0.07
Medicare	66.42	67.19	67.16	
Medicaid	5.71	6.03	6.02	
Private	24.75	23.13	23.18	
Others ^||^	3.11	3.65	3.64	
Median household income in quartile				0.07
1st	24.95	27.25	27.18	
2nd	22.80	24.41	24.36	
3nd	25.24	24.51	24.53	
4th	27.01	23.84	23.93	
Hospital region				0.05
Northeast	22.04	22.35	22.34	
Midwest	25.23	22.79	22.87	
South	35.72	38.36	38.28	
West	17.01	16.50	16.51	

	Multiple Myeloma		#ASD
	Yes	No	
No. of observation, weighted	3273	3318	
Age categories			0.09
18-49	5.38	5.76	
50-59	14.36	15.98	
60-69	31.16	28.50	
70-79	30.13	28.71	
≥ 80	18.97	21.05	
Female	48.64	48.69	0.00
Race/Ethnicity			0.05
White	67.31	67.61	
Black	20.99	21.12	
Hispanic	7.27	6.99	
#Others	4.43	4.28	
Comorbidities			
Dyslipidemia	26.09	25.64	0.01
Atrial fibrillation	21.84	21.53	0.01
Chronic obstructive pulmonary disease	17.51	17.66	0.00
Hypertension	60.10	59.95	0.00
Peripheral vascular disease	5.44	5.52	0.00
Stem cell transplantation	13.84	13.92	0.00
Neutropenia	12.74	12.85	0.00
Chemotherapy	7.82	7.63	0.01
Inflammatory bowel disease	4.43	4.43	0.00
Diabetes	25.76	25.70	0.00
Obesity	7.42	7.54	0.00
Deficiency anemia	41.19	40.94	0.01
Congestive heart failure	21.81	21.92	0.00
Chronic renal disease	38.89	38.30	0.01
Liver disease	2.17	2.24	0.01
Smoking	18.45	18.45	0.00
Elixhauser score			0.00
0	4.55	4.62	
1 - 3	25.73	26.04	
≥ 4	69.72	69.33	
Hospital bed size			0.03
Small	11.34	11.68	
Medium	22.12	21.91	
Large	66.54	66.41	
Hospital teaching status			0.05
Rural	5.41	5.90	
Urban, non-teaching	29.48	29.74	
Urban, teaching	65.11	64.36	
Expected primary payer			0.00
Medicare	67.74	67.69	
Medicaid	5.47	5.48	
Private	23.93	24.08	
Others ^||^	2.87	2.75	
Median household income in quartile			0.03
1st	25.33	25.45	
2nd	22.40	22.53	
3nd	24.87	24.82	
4th	27.41	27.19	
Hospital region			0.08
Northeast	22.71	23.57	
Midwest	22.81	20.18	
South	37.26	39.20	
West	17.22	17.04	

The result following propensity-derived inverse probability weighting is shown in Table 3. We checked the balance of the baseline characteristics between the two groups. A balance of more than 0.1 of the standardized difference between the two groups was considered significant [17]. The inverse probability weighting achieved similar baseline characteristics between the two groups (Table 3).

**Table 3:** Inverse Probability Weighting of Baseline Characteristics of Participants with Multiple Myeloma and Clostridium difficile (C. diff) in the United States #ASD: absolute standardized differences

	Multiple Myeloma		#ASD
	Yes	No	
No. of observation, weighted	3,273	3,318	
Age categories			0.09
18-49	5.38	5.76	
50-59	14.36	15.98	
60-69	31.16	28.50	
70-79	30.13	28.71	
≥ 80	18.97	21.05	
Female	48.64	48.69	0.00
Race/Ethnicity			0.05
White	67.31	67.61	
Black	20.99	21.12	
Hispanic	7.27	6.99	
#Others	4.43	4.28	
Comorbidities			
Dyslipidemia	26.09	25.64	0.01
Atrial fibrillation	21.84	21.53	0.01
Chronic obstructive pulmonary disease	17.51	17.66	0.00
Hypertension	60.10	59.95	0.00
Peripheral vascular disease	5.44	5.52	0.00
Stem cell transplantation	13.84	13.92	0.00
Neutropenia	12.74	12.85	0.00
Chemotherapy	7.82	7.63	0.01
Inflammatory bowel disease	4.43	4.43	0.00
Diabetes	25.76	25.70	0.00
Obesity	7.42	7.54	0.00
Deficiency anemia	41.19	40.94	0.01
Congestive heart failure	21.81	21.92	0.00
Chronic renal disease	38.89	38.30	0.01
Liver disease	2.17	2.24	0.01
Smoking	18.45	18.45	0.00
Elixhauser score			0.00
0	4.55	4.62	
1 - 3	25.73	26.04	
≥ 4	69.72	69.33	
Hospital bed size			0.03
Small	11.34	11.68	
Medium	22.12	21.91	
Large	66.54	66.41	
Hospital teaching status			0.05
Rural	5.41	5.90	
Urban, non-teaching	29.48	29.74	
Urban, teaching	65.11	64.36	
Expected primary payer			0.00
Medicare	67.74	67.69	
Medicaid	5.47	5.48	
Private	23.93	24.08	
Others ^||^	2.87	2.75	
Median household income in quartile			0.03
1st	25.33	25.45	
2nd	22.40	22.53	
3nd	24.87	24.82	
4th	27.41	27.19	
Hospital region			0.08
Northeast	22.71	23.57	
Midwest	22.81	20.18	
South	37.26	39.20	
West	17.22	17.04	

After a multivariate analysis, the following independent predictors were found to be associated with the rate of CDI: female gender (OR: 1.13; 95% confidence interval (CI): 1.04 - 1.23, p = 0.003), chronic renal disease (OR: 1.19; 95% CI: 1.09 - 1.30, p < 0.0001), stem cell transplant (OR: 1.39; 95% CI: 1.24 - 1.56, p < 0.0001), neutropenia (OR 1.77; 95% CI: 1.57 - 2.00, p < 0.0001), inflammatory bowel disease (OR 1.68, 95% CI: 1.40 - 2.10, p < 0.0001), and atrial fibrillation (OR 1.24, 95% CI: 1.12 - 1.37, p < 0.0001). Comorbid conditions were assessed using the ECI categories: ECI 1 - 3 (OR 1.47, 95% CI: 1.23 - 1.76, p < 0.0001) and category ECI ≥ 4 (OR 2.64, 95% CI: 2.19 - 3.19, p < 0.001) (Table 4).

**Table 4:** Odds Ratio for Clostridium Difficile Infections Among Multiple Myeloma Patients with Various Conditions and Status aOR: adjusted odds ratio; CI: confidence interval

Variables	aOR	CI (95%)		P value
Year				
2010 (Ref.)				
2011	0.92	0.71	1.17	1.000
2012	1.14	0.92	1.43	1.000
2013	1.00	0.79	1.25	1.000
2014	1.11	0.88	1.39	1.000
2015	1.10	0.87	1.39	1.000
Age categories				
18-49 (ref.)				
50-59	0.88	0.65	1.18	1.000
60-69	1.01	0.75	1.35	1.000
70-79	0.94	0.69	1.27	1.000
≥ 80	0.77	0.56	1.07	0.242
Race/Ethnicity				
White (ref.)				
Black	0.92	0.79	1.06	0.657
Hispanic	0.90	0.73	1.11	1.000
#Others	0.86	0.66	1.12	0.775
Female	1.13	1.04	1.23	0.003
Obesity	0.86	0.75	1.00	0.048
Peripheral vascular disease	1.04	0.88	1.24	0.645
Chronic renal disease	1.19	1.09	1.30	
Atrial fibrillation	1.24	1.12	1.37	
Stem cell transplantation	1.39	1.24	1.56	
Neutropenia	1.77	1.57	2.00	
Chemotherapy	0.96	0.83	1.11	0.613
Inflammatory bowel disease	1.68	1.40	2.10	
Congestive heart failure	0.99	0.89	1.09	0.819
Hypertension	0.82	0.75	0.89	
Chronic liver failure	0.84	0.65	1.08	0.178
Elixhauser score				
0 (ref.)				
1 - 3	1.47	1.23	1.76	
≥ 4	2.64	2.19	3.19	
Hospital bed size				
Small (ref.)				
Medium	1.03	0.85	1.25	1.000
Large	1.17	0.99	1.39	0.065
Hospital teaching status				
Rural (ref.)				
Urban, non-teaching	1.21	0.95	1.54	0.190
Urban, teaching	1.34	1.06	1.70	0.003
Expected primary payer				
Medicare (ref.)				
Medicaid	1.03	0.85	1.24	1.000
Private	1.07	0.92	1.24	1.000
Others ^||^	0.86	0.61	1.20	1.000
Median household income in quartile				
1^st^ (ref.)				
2nd	1.01	0.87	1.18	1.000
3nd	1.07	0.91	1.26	1.000
4th	1.16	0.99	1.36	0.069

Table 5 shows the impact of CDI on the clinical outcomes of MM patients. Patients with CDI had 67% increased odds of death in hospitalized patients compared with those without CDI (OR: 1.67; 95% CI: 1.49 - 1.88, p < 0.0001). In addition, there was significantly increased odds of AKI (OR: 1.35; 95% CI: 1.26 - 1.45, p < 0.0001) and AKI requiring hemodialysis (OR: 6.20 vs. 3.84, 95% CI: 1.42 - 1.92, p < 0.0001) among patients with CDI. Patients with CDI were also more likely to have higher healthcare resource utilization with significantly higher costs of hospitalization ($32,862 vs. $20,257, mean ratio (MR): 1.62; 95% CI: 1.54 - 1.70, p < 0.0001, and more prolonged length of stay (12.61 vs. 8.00, MR: 1.67; 95% CI: 1.52 - 1.64, p < 0.0001).

**Table 5:** Clinical Outcomes of Multiple Myeloma (MM) in Clostridium Difficile Infection (CDI) Patients ¶ represents odds ratio (with 95% CI) of C. diff based on propensity score inverse probability weighing *Average cost ratio MR: ¥ Mean ratio **Average length of stay ratio CI: confidence interval; MR: mean ratio; OR: odds ratio

	C. diff		OR¶/¥MR (95% CI) P-value	
	Yes	No	
Inpatient mortality, %	9.85	6.13	1.67 (1.49, 1.88) < 0.0001
Acute kidney injury, %	36.24	29.59	1.35 (1.26, 1.45) < 0.0001
Acute kidney Injury requiring dialysis, %	6.20	3.84	1.66 (1.42, 1.92) < 0.0001
Cost, average*	32,862	20,257	1.62 (1.54, 1.70) < 0.0001
Length of stay, average **	12.61	8.00	1.58 (1.52, 1.64) < 0.0001

Table 6 shows the trend analysis. We observed signiﬁcant changes in the rates of CDI and CDI mortality in MM patients with or without adjustment for demographics, comorbidities, and hospital characteristics. CDI incidence appears to have increased over the years (average rate of 3.27% per year), while the mortality rate is in the opposite direction with an average of 10% decrease per year).

**Table 6:** Trend of Clostridium Difficile Infection (CDI) in Multiple Myeloma Patients

	2010	2011	2012	2013	2014	P-value for trend (average change per year)
CDI (crude rate)	2.95%	2.85%	3.10%	2.98%	3.46%	0.016 (+4.02%)
CDI (adjusted rate)	2.19%	2.05%	2.45%	2.20%	2.48%	0.048 (+3.27%)
Mortality in CDI	16.23%	19.69%	13.44%	15.52%	10.90%	0.008 (-10.03%)

## Discussion

We report results from a large database on CDI incidence and CDI-related complications in hospitalized MM patients over a five-year period. CDI is the most common cause of healthcare-associated infections in US hospitals [1]. Our study revealed an average incidence of 3.1% CDI in MM patients and this incidence increased from 2.95% in 2010 to 3.46% in 2014. We found that in addition to the well-established risk factors for CDI, such as female gender, CKD, HSCT, neutropenia, and inflammatory bowel disease, AF was also significantly associated with CDI in MM patients. CDI risk also increased with increased comorbidity index and connoted worse clinical outcomes.

Compared to the non-oncology population, CDI is more common in oncology patients [18]. The cumulative CDI rate in our study on MM is consistent with prior studies, although other studies reported a lower incidence in lymphoma patients [4] and a higher incidence in leukemia patients [19]. This discrepancy may reflect differences in risk based on the type of hematologic malignancy. The higher incidence of CDI in oncology patients is reported to be related to prolonged antibiotic use, chemotherapy, and prolonged proton pump inhibitor use [18-20]. Our study, however, did not find an association between chemotherapy and CDI in hospitalized MM patients. It is worth noting that the well-documented chemotherapeutic agents, such as cisplatin, etoposide, bleomycin, paclitaxel, vinblastine, 5-fluorouracil, cyclophosphamide, methotrexate, doxorubicin, and cytarabine-based regimens, are not the mainstay treatment agents in MM [21-22]. Immunomodulatory drugs used in MM stimulate T-cells and natural killer cells, thereby enhancing the immune response (anti-MM immune activity) and potentially may attenuate the risk of CDI [21]. Our finding of an increased association between CDI and CKD is consistent with previous studies. In a national cohort of hospitalized patients, there was a two-fold higher risk of CDI in CKD patients with end-stage renal disease (ESRD); patients requiring dialysis are at the highest risk [23]. Patients with CKD are particularly vulnerable to infection [23-24], given its immunosuppressive effects. Besides, CKD patients are also at a higher risk of prolonged hospital stay and antibiotic exposure [25]. We also demonstrated that MM patients with AF have a higher incidence of CDI. Hospitalized patients with cardiac co-morbidity, such as chronic heart failure, were found to have an increased risk of acquiring CDI [26]. AF, as an independent risk associated with CDI, has recently been identified by another study [27]. Cardiovascular diseases with the inclusion of AF through hypoperfusion phenomena cause bowel ischemia or metabolic change in patients with CDI, which potentially increases the risk of bacterial translocation and alteration of intestinal flora leading to CDI.

CDI is a well-recognized nosocomial infection and accounts for almost 15% to 25% of all cases of nosocomial diarrhea; up to 35% of patients develop recurrent CDI after the initial treatment [28]. Our data show that patients with MM and CDI had worse outcomes compared to those without CDI. Several hospital-based studies have previously demonstrated that CDI in hospitalized patients has been associated with adverse outcomes and increased mortality [19, 29]. Inpatient mortality rates from CDI as high as 4.2% - 6.9% were found in several centers in North America. We found a 9.9% mortality among patients with CDI and a 67% increased odds of death compared to those without CDI. Patients with CDI had a higher inpatient mortality rate and a longer length of hospital stay, resulting in higher hospital costs. In addition to increased mortality among CDI patients, we found an increased risk of acute kidney injury (AKI) and AKI requiring dialysis, which plays a profound role in prolonged hospitalization, hospital costs, and potentially increased mortality in these patients. In addition to volume depletion from prolonged diarrhea, C. diff toxins induced cell death and inflammation. It is, therefore, not surprising that AKI has been recognized by the Infectious Diseases Society of America as a marker of severe CDI. Strategies directed at preventing AKI in CDI patients may, therefore, represent a potential target at improving their outcomes. In a single-center study, patients with Stage 4 to 5 CKD had a significant risk of developing CDI [30]. In our study, we did not subclassify CKD patients according to their eGFR and stratify their association with CDI in MM patients. However, we believe it would be valuable to do so in the future to improve management in the outcome of MM patients with CDI.

Despite the increase rate the mortality rate caused by CDI trends down, it could be due to the better recognition and treatment. In addition, direct using a new diagnostic approach, e.g., polymerase chain reaction (PCR), may cause overdiagnosis of inpatient CDI.

Our study has some limitations that deserve to be emphasized. First, in the NIS database, variables are identified using a coding system that is subject to coding errors and documentation disparities. Second, this analysis did not permit us to directly ascribe the associations to be causative for CDI; however, it did identify the more vulnerable population for CDI and provided a rationale for the development of more effective approaches to preventing CDI. Third, our study was limited to the in-hospital outcomes; follow-up data were not reported. Additionally, the ICD-9-CM does not code for long-term antibiotic use, and hence we could not identify these patients. We also could not identify the prolonged proton pump inhibitor use and the utility of immunomodulatory medications. However, the strengths of this study included the nationally-representative large sample size which involved multiple centers and populations across the US. Lastly, this study, by design, provides data that support strongly associated risk factors between CDI and MM but not a causal relationship.

## Conclusions

In conclusion, we found that increased inpatient mortality and prolonged hospitalization, as well as costs in MM patients with CDI, compared to those without CDI. We also identified HSCT, neutropenia, inflammatory disease, AF, and CKD as associated risk factors for CDI in these patients. The results provide a rationale for the development of more effective approaches to preventing healthcare-associated CDI in MM patients.
